# Disruption of *OVOL2* Distal Regulatory Elements as a Possible Mechanism Implicated in Corneal Endothelial Dystrophy

**DOI:** 10.1155/2024/4450082

**Published:** 2024-01-04

**Authors:** Lubica Dudakova, Lenka Noskova, Stanislav Kmoch, Martin Filipec, Ales Filous, Alice E. Davidson, Vasileios Toulis, Jana Jedlickova, Pavlina Skalicka, Hana Hartmannova, Viktor Stranecky, Jana Drabova, Drahuse Novotna, Marketa Havlovicova, Zdenek Sedlacek, Petra Liskova

**Affiliations:** ^1^Research Unit for Rare Diseases, Department of Paediatrics and Inherited Metabolic Disorders, First Faculty of Medicine, Charles University and General University Hospital in Prague, Ke Karlovu 2, 128 08 Prague, Czech Republic; ^2^Department of Ophthalmology, First Faculty of Medicine, Charles University and General University Hospital in Prague, U Nemocnice 2, 128 08 Prague, Czech Republic; ^3^Department of Ophthalmology, Second Faculty of Medicine, Charles University and University Hospital Motol, V Úvalu 84/1, 150 06 Prague, Czech Republic; ^4^UCL Institute of Ophthalmology, EC1V 9EL London, UK; ^5^Department of Biology and Medical Genetics, Second Faculty of Medicine, Charles University and University Hospital Motol, V Úvalu 84/1, 150 06 Prague, Czech Republic

## Abstract

The genetic architecture of corneal endothelial dystrophies remains unknown in a substantial number of affected individuals. The proband investigated in the current study was diagnosed in the neonatal period with bilateral corneal opacification due to primary endothelial cell dysfunction. Neither his parents nor his sister had signs of corneal disease. Conventional karyotyping revealed a *de novo* translocation involving chromosomes 3 and 20, t(3;20)(q25;p11-12). Following genome and targeted Sanger sequencing analysis, the breakpoints were mapped at the nucleotide level. Notably, the breakpoint on chromosome 20 was identified to lie within the same topologically associated domain (TAD) as corneal endothelial dystrophy-associated gene *OVOL2*, and it is predicted to disrupt distal enhancers. The breakpoint at chromosome 3 is located within intron 2 of *PFN2*, which is currently not associated with any human disease. Further interrogation of the proband's genome failed to identify any additional potentially pathogenic variants in corneal endothelial dystrophy-associated genes. Disruption of a candidate *cis*-regulatory element and/or positional effects induced by translocation of *OVOL2* to a novel genomic context may lead to an aberrant *OVOL2* expression, a previously characterized disease mechanism of corneal endothelial dystrophy. Further research is necessary to explore how disruption of regulatory elements may elucidate genetically unsolved corneal endothelial dystrophies.

## 1. Introduction

Bilateral congenital corneal edema manifesting as opacity with milky appearance can be a sign of three different inherited endothelial diseases: congenital hereditary endothelial dystrophy (CHED), posterior polymorphous corneal dystrophy (PPCD), and X-linked endothelial corneal dystrophy [1–5].

PPCD is an autosomal-dominant disease, typically manifesting as the presence of vesicular lesions, bands, and opacities at the level of the Descemet membrane and the corneal endothelium [2, 6–8]. Around one-third of patients develop corneal endothelial edema requiring grafting in order to restore vision. Typically, endothelial failure occurs in PPCD later in life; however, cases suffering from bilateral congenital edema have also been reported [4, 5, 7]. The disease mechanism involves dysregulation of epithelial-mesenchymal transition (EMT) and its converse mechanism, mesenchymal-to-epithelium transition (MET). To date, variants in three genes, zinc finger E-box binding homeobox 1 (*ZEB1*, OMIM ^∗^189909), ovo-like zinc finger 2 (*OVOL2*, OMIM ^∗^616441), and grainy head-like 2 (*GRHL2*, OMIM ^∗^608576), encoding three mutually regulated transcription factors (TFs), have been identified to cause PPCD [7–10]. While pathogenic loss-of-function (LoF) *ZEB1* variants underlie PPCD3 [3, 9], all disease-associated variants in *OVOL2* and *GRHL2* reported to date affect regulatory regions resulting in ectopic expression of the encoded proteins in the corneal endothelium causing PPCD1 and PPCD4, respectively [7, 8, 10].

In CHED, which is inherited as an autosomal recessive trait, congenital corneal opacification and marked corneal thickening are pathognomonic [11, 12]. Another feature is hearing loss which has been reported to develop variably with time [13–15]. The disease is caused by primary corneal endothelium dysfunction due to biallelic mutations in the solute carrier family 4 member 11 gene (*SLC4A11*; OMIM ^∗^610206) encoding a cell adhesion molecule and a transmembrane protein carrier regulating water flux [16–19]. Finally, the X-linked endothelial corneal dystrophy has been linked to Xq25. The disease-causing gene is yet to be identified [1].

*cis*-Regulatory elements (CREs) typically comprise short DNA sequences (100 to 500 bp) that serve as binding recognition sites for TFs recruiting transcriptional machinery to specific sites in the genome [20]. Genetic variation in CREs can alter TFs' binding capacity and thus dysregulate the linked genes, which may consequently lead to abnormal phenotypes [7, 8]. It has been shown that regulatory elements can be located up to 1 Mb away from the gene that they regulate [21].

Topologically associated domains (TADs) are structurally distinct functional units of chromatin within which interactions between genes and CREs occur, while interactions across different TADs are far less abundant [22]. The mechanisms of TAD function and the nature of their boundaries are not fully understood, but it is known that TAD disruption can affect the expression of nearby genes [23, 24].

Despite recent advances, some corneal endothelial dystrophy patients remain genetically unsolved suggesting further genetic heterogeneity [25].

In this study, we report a sporadic patient with bilateral congenital corneal endothelial dystrophy and a *de novo* reciprocal chromosomal translocation with one breakpoint downstream of the *OVOL2* gene, which is predicted to disrupt the TAD structure on chromosome 20 (chr20) and disturb the genomic architecture of *OVOL2* regulatory elements.

## 2. Material and Methods

### 2.1. Study Subjects and Clinical Examination

The study followed the tenets of the Declaration of Helsinki and was approved by the Ethics Committee of the General University Hospital in Prague (151/11 S-IV). Informed consent was obtained from the study participants prior to the enrolment.

A standard ophthalmic examination was performed in the proband, his parents, and sister. Best corrected visual acuity (BCVA) was determined using Snellen charts and noted in decimal values. Corneal diameter, corneal curvature, anterior chamber depth, and axial length were measured with an IOLMaster 500 (Carl Zeiss Meditec, Jena, Germany). Corneal thickness was determined using high-resolution spectral domain optical coherence tomography (SD-OCT) (Spectralis; Heidelberg Engineering, Heidelberg, Germany). Specular microscope Noncon ROBO Pachy SP-9000 (Konan Medical Inc., Tokyo, Japan) was used to visualize the cornel endothelium in detail.

### 2.2. DNA Extraction, Karyotyping, and Paternity/Maternity Testing

Genomic DNA was extracted from lymphocytes using a Gentra Puregene Blood Kit (Qiagen, Hilden, Germany) or from buccal cells using an Oragene Saliva Collection and DNA Extraction Kit (Genotek, Ottawa, Canada), according to the manufacturer's instructions. Conventional karyotyping was performed in the proband and his parents. Standard paternity and maternity testing using short tandem repeat markers was also conducted [26].

### 2.3. Genome Sequencing

Genome sequencing was performed in the proband with a TruSeq Nano DNA Library Preparation Kit (Illumina, San Diego, CA, USA) on a HiSeq X-Ten sequencer (Illumina). Generated 150 bp pair-end reads were aligned to the human reference genome (hg38) with NovoAlign (Novocraft, Malaysia). The mean depth of coverage was >40x. Variants were called using the Genome Analysis Toolkit 4 HaplotypeCaller [27]. Annotation was performed with the Variant Effect Predictor, and allele frequencies were retrieved from the Genome Aggregation Database (gnomAD) v3.1.1 providing sequencing data from 76,156 genomes [28].

### 2.4. Identification and Confirmation of Translocation Breakpoints

Translocation breakpoints and copy number variants (CNVs) were assessed in the genome data using three different tools: Lumpy [29], Delly [30], and Manta [31]. Reads were visualized in the Integrative Genomic Viewer (IGV) [32], and the sequence of the breakpoints was analyzed from split and discordant reads. Conventional Sanger sequencing was employed to resolve the breakpoint junctions at the nucleotide level in the proband and to confirm their absence in his parents and sister. For this purpose, two sets of PCR primer pairs were designed, BPA2F (5′ CACGTTGGTGCAGGTCATAC 3′) and BPA2R (5′ CCCTTGAGTGTAGGCTGGAC 3′) for amplification across the junction on the derivative chr3 (der3) and BPB2F (5′ AAAAGGCATGGAGAGCTGAA 3′) and BPB2R (5′ AGTTTTGGCCACACACACAA 3′) for amplification on the derivative chr20 (der20). The PCR products were analyzed using gel electrophoresis, and then, they were purified and sequenced on an ABI 3700 sequencer (Applied Biosystems, Foster City, CA, USA).

### 2.5. *In Silico* Analysis of Possible Impacts of the Translocation on Gene Expression

The UCSC Genome Browser (https://genome.ucsc.edu) was used to determine whether the translocation breakpoints lie within annotated gene regions. Possible regulatory effects were assessed using publicly available TAD datasets. As corneal endothelial cell-specific Hi-C data is currently not available, data from H1 NPCs—neural progenitor cells generated from the H1 human embryonic stem cell (ESC) line—and H9 ESC-derived neuroectodermal cells was interrogated as a proxy. The data can be found in the 3D Genome Interaction Viewer and Database (3DIV, http://kobic.kr/3div/) [33]. Hi-C datasets were analyzed using two algorithms, DI (with a window size of 2 Mb) [22] and TopDom (with a window size of 5 Mb) [34], and the results were compared.

Data from the Encyclopedia of DNA Elements (ENCODE) and GeneHancer databases were then used to ascertain the presence of CREs in the disrupted regions and their inferred target genes [35, 36].

Finally, comparisons with deletion, duplication, and inversion breakpoints observed in healthy individuals were performed using the Database of Genomic Variants (DGV) and gnomAD SVs v2.1 [37, 38].

### 2.6. Analysis of Variants in Known Genes for Corneal Diseases

To exclude the possible role of variants in other genes, rare variants with minor allele frequency (MAF) ≤ 0.0001 at genomic loci known to be associated with endothelial dystrophies that may manifest as congenital or childhood-onset corneal edema, i.e., *OVOL2*, *GRHL2*, *ZEB1*, and *SLC4A11* [39, 40] (including regions ≤ 1 Mb from the transcription start and end), were subjected to further evaluation. The MAF threshold was chosen based on the rarity of congenital corneal opacity in the general population [41].

## 3. Results

### 3.1. Clinical Assessment

The proband with no family history of corneal dystrophy was noted to be photophobic since birth. At 2 months of age, milky appearance of both corneas was noted. He was examined under general anesthesia at the age of 4 months, and a diagnosis of corneal endothelial dystrophy was made. The parents reported that the corneal opacification partially resolved by the age of 3 years.

**Figure 1:**
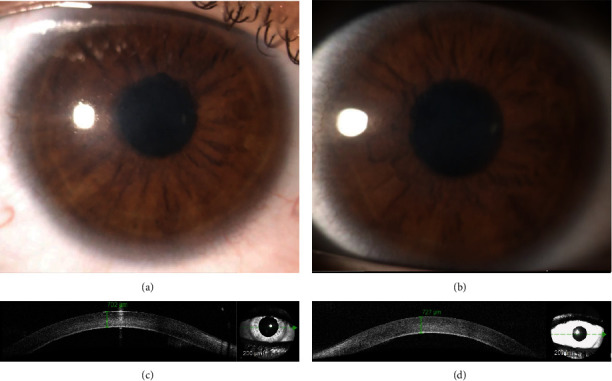
Anterior segment findings in the 12-year-old proband. Opacification of the normally clear right (a) and left (b) cornea can be observed. SD-OCT imaging shows increased central corneal thickness and hyperreflexia of the posterior corneal layers in the right (c) and left (d) eyes.

At 7 years, he had a visual loss with BCVA decreased to 0.33 in both eyes. Aged 12, he was noted to have exotropia and moderate vision impairment with BCVA 0.3 in the right eye and 0.4 in the left eye. Both corneas were hazy precluding specular microscopy imaging to visualize the corneal endothelium. Only one image was considered partially informative (Supplementary Figure 1). Central corneal thickness was 702 *μ*m and 727 *μ*m in the right and left eyes, respectively (Figure 1) (normal value is 521.3 ± 44.7 *μ*m) [42]. Axial length was 22.18 mm in the right eye and 21.86 mm in the left eye (average value in 15-year-old male children of European descent is 23.41 ± 0.86 mm) [43]. Keratometry readings (K1/K2) were 43.05/44.06 diopter (D) in the right eye and 45.06/46.62 D in the left eye (normal values are 43.53 ± 1.49 D and 44.28 ± 1.52 D for K1 and K2, respectively) [44]. Intraocular pressure corrected for increased corneal thickness was bilaterally within the normal range. Fundus examination and SD-OCT of the macular region did not reveal any pathology. The retinal nerve fiber layer thicknesses were bilaterally also within the normal range, excluding glaucomatous changes.

The proband did not have a hearing impairment, and his development by the age of 16 was normal. His parents and sister had no signs of endothelial corneal dystrophy.

### 3.2. Mapping of the Translocation Breakpoints and Their Gene Content

**Figure 2:**
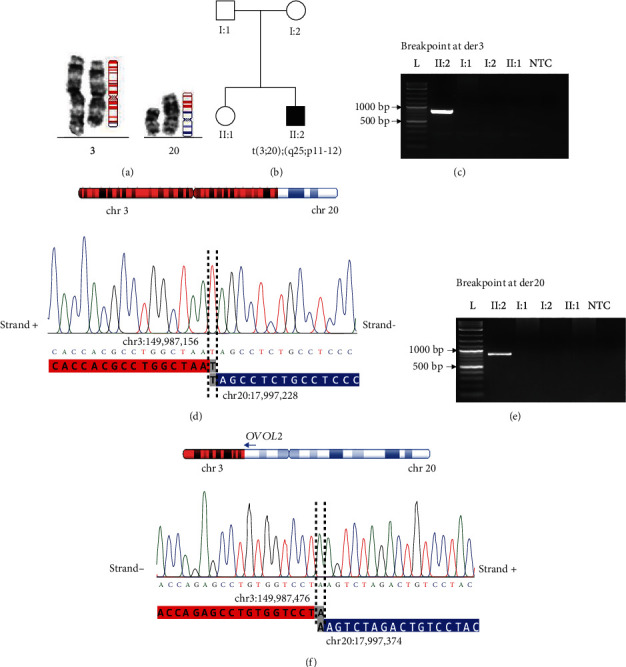
Results of genetic analysis of the proband. (a) Partial karyotype showing the reciprocal translocation between chr3 and chr20. The schematic representation illustrates parts of der3 and der20 originating from chr3 (red) and chr20 (blue). (b) The pedigree of the family. Images of gel electrophoresis of PCR products amplified across the breakpoint junctions on (c) der3 (850 bp) and (e) der20 (947 bp). NTC: no template control. Sanger sequencing of PCR product showing the details of the junctions including the 1 bp microhomologies at (d) chromosome 3 and (f) chromosome 20. The coordinates (hg38) mark nucleotides participating in the microhomologies.

Karyotyping revealed a *de novo* (both paternity and maternity were confirmed) reciprocal translocation t(3;20)(q25;p11-12) in the proband. The translocation breakpoints were finely mapped using genome sequencing and confirmed by PCR and Sanger sequencing across the junctions on der3 and der20. Human build hg19 and hg38 coordinates of the breakpoints are provided in Supplementary Table 1. The translocation was not fully balanced (with a loss of 319 bp and 145 bp from chr3 and chr20, respectively). Both junctions showed 1 bp microhomologies (the lengths of the regions lost indicated above correspond to the situation when the microhomology at one junction originates from chr3 and at the other junction from chr20) (Figure 2 and Supplementary Figure 2).

The breakpoint on chr20 was located in an intergenic region ~27 kb downstream of the *OVOL2* stop codon (NM_021220.4) and ~6 kb downstream of the stop codon of the *MGME1* gene (NM_052865.4). The breakpoint on chr3 was located in intron 2 of the *PFN2* gene (NM_053024.4).

The newly juxtaposed genes or gene fragments were positioned in antisense orientation on both derivative chromosomes, which were thus unlikely to produce any chimeric transcripts. The structural variants can be described according to the current nomenclature [45] as follows: NC_000003.12:g.qter_149987157delins[NC_000020.12:g.pter_17997228] and NC_000020.12:g.pter_17997375delins[NC_000003.12:g.qter_149987476]. No translocations or deletions similar to those adjacent to the translocation breakpoints were found in datasets of a normal human genetic variation (gnomAD SVs v2.1 and DGV).

### 3.3. Possible Regulatory Impacts of the Translocation

**Figure 3:**
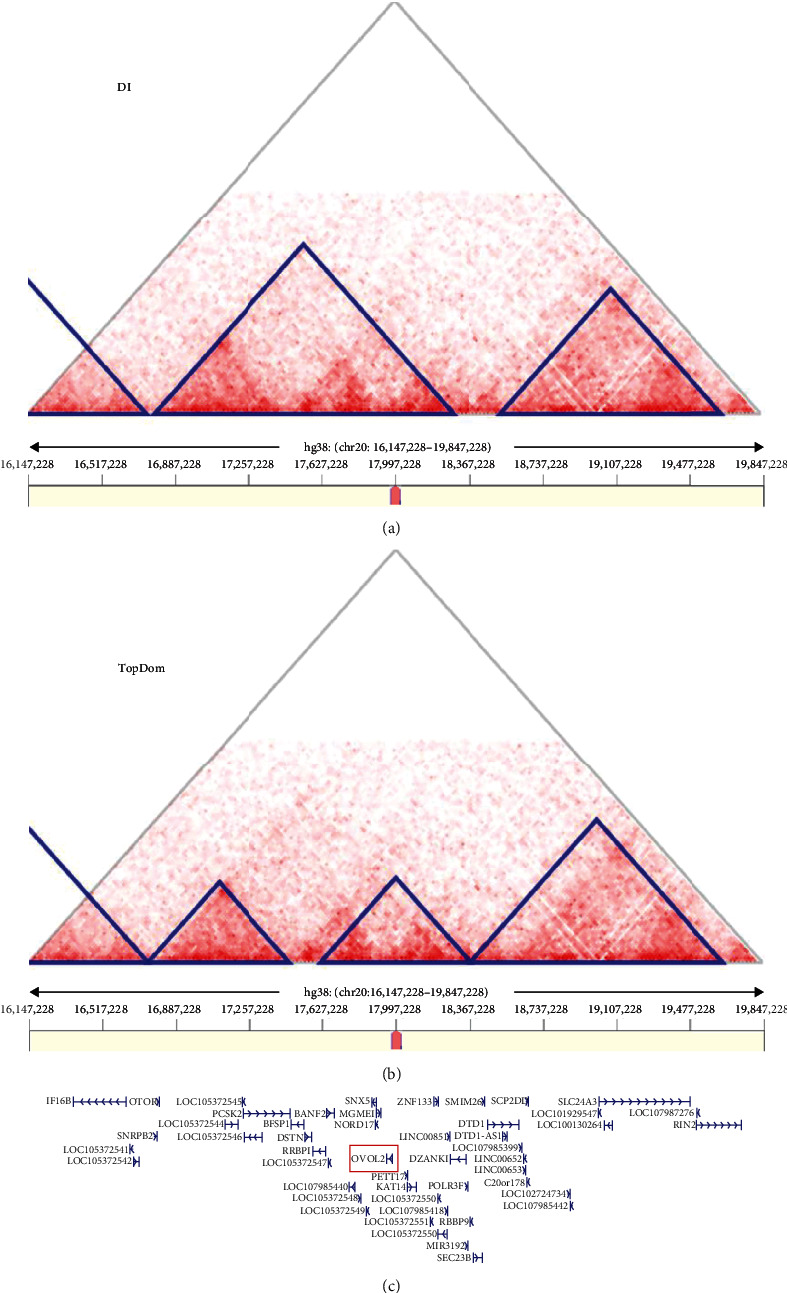
Visualization of topologically associated domains (TADs) at chr20p11.23 in neural progenitor cells (H1 NPCs). Interaction heat maps, in which TADs are indicated as triangles using (a) DI and (b) TopDom algorithms. The position of the breakpoint is highlighted with a red bar. (c) Gene annotation (MANE transcripts), *OVOL2* is shown embedded in a red box.

For the TAD analysis, we have selected data generated from H1 NPCs and H9 ESC-derived neuroectodermal cells as the corneal endothelium is of cranial neural crest cell origin, and data directly from the corneal endothelium is not currently available [46]. The analysis of the TAD structure of the chr20 breakpoint regions using the TopDom algorithm indicated that the breakpoint at chr20 disrupted a TAD in both cell lines analyzed, and *OVOL2* was always positioned within the same TAD. The DI algorithm showed also that the breakpoint affects a TAD in both cell lines and that *OVOL2* and the breakpoint were located within the same TAD (Figure 3). Regarding the breakpoint at chr3, both algorithms indicated disruption of a TAD in both cell lines. An example of the analysis is shown in Figure 3, and complete data are presented in Supplementary Figures 3–5.

**Figure 4:**
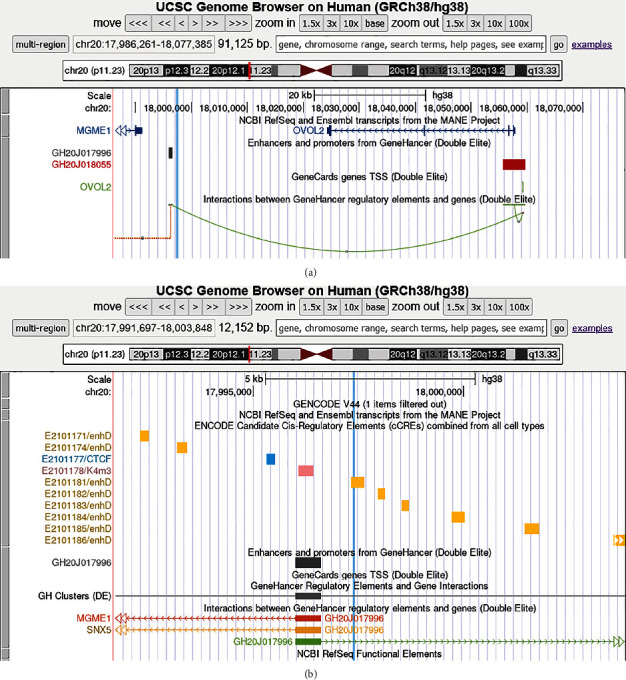
Visualization of interactions around the position of chromosomal break on chr20. (a) View of the translocation region showing GeneHancer data including the position of enhancer ID GH20J017996 (grey rectangle) which is predicted to interact with the *OVOL2* promoter GH20J018055 (red rectangle). Light blue vertical line indicates the location of the breakpoint. (b) More detailed view of the region showing also ENCODE candidate *cis*-regulatory elements (note the direct interruption of EH38E2101181) in addition to the GeneHancer data. Light blue vertical line indicates the location of the breakpoint. Yellow rectangles represent a distal enhancer-like signature; blue rectangle, the CTCF binding site; and pink rectangle, the DNase-H3K4me3 mark.

If *OVOL2* is within the same TAD as the breakpoint, the der20 may lack some important CREs downstream of *OVOL2* that may induce aberrant gene expression. According to the GeneHancer database, a high-confidence enhancer (GH20J017996) which normally interacts with the *OVOL2* promoter (GH20J018055) is separated from *OVOL2* by the translocation (Figure 4(a)). According to ENCODE, there are several other candidate regulatory regions and CTCF binding sites in the region of chr20 separated by the translocation from *OVOL2*, and a candidate distal enhancer of *OVOL2*, EH38E2101181, is directly interrupted by the breakpoint (Figure 4(b)).

The breakpoint at chr3 disrupted the *PFN2* gene (OMIM ^∗^176590), which is not known to be associated with any human disease. The gene has a pLI score of 0.42 indicating tolerance to LoF variants [47].

### 3.4. Exclusion of Causal Variants in Known Genes

We also evaluated the possible pathogenicity of variants in genes known to be associated with congenital or childhood-onset monogenic corneal endothelial dystrophies (i.e., *GRHL2*, *ZEB1*, and *OVOL2*). Six rare heterozygous variants (MAF ≤ 0.0001) were identified in the patient (Supplementary Table 2). All were located in noncoding regions, and one was unique to the family, i.e., not present in gnomAD v.3.1.1. However, segregation analysis showed that five of the six variants were inherited from one of the unaffected parents and, hence, unlikely to be responsible for an autosomal-dominant disease. The private rare *SLC4A11* variant, c.217+1131A>G, occurred in the proband *de novo*; however, *in silico* analysis did not support its likely involvement in aberrant splicing nor was it located within any annotated regulatory elements. Furthermore, congenital corneal opacity is attributed to biallelic mutations in *SLC4A11*, and dysregulation of *SLC4A11* due to translocation is unlikely given the large genomic distance of the gene from the breakpoint on chr20 (~14 Mb); hence, the heterozygous variant alone was also deemed highly unlikely to be disease-causing in the proband.

## 4. Discussion

In this study, we performed comprehensive genomic characterization of a patient with primary endothelial dysfunction due to congenital corneal edema. We identified that the affected male carried a *de novo* reciprocal chromosomal translocation t(3;20)(q25;p11-12). One of the breakpoints was positioned directly downstream of the endothelial dystrophy-associated gene *OVOL2*, which prompted us to focus on this structural variant as possibly disease-causing.

It has been previously demonstrated that variants altering *OVOL2* CREs give rise to PPCD1 which may manifest similarly to the case reported in this study as congenital corneal edema [7, 48]. On this basis, we hypothesize that the reciprocal translocation may induce ectopic *OVOL2* expression in corneal endothelial cells and explain the phenotypic outcome in the proband. This is further supported by the *de novo* origin of the translocation and sporadic occurrence of the disorder in the family.

Chromosomal rearrangements are known to influence gene expression either by direct disruption of genes located at the breakpoints (which may lead to gene inactivation or formation of fusion genes) or by positional effects in which genes located in the vicinity of the breakpoints are removed from their normal CREs or are moved to regions under the control of foreign elements [49].

Fine mapping using genome sequencing showed that in our patient, the breakpoint is located ~27 kb downstream of *OVOL2*. Analysis of TADs in two cell lines of the most similar embryonic origin available indicated that the translocation is likely to disrupt the normal TAD structure around *OVOL2* and that it could possibly create a neo-TAD, resulting in aberrant expression of the gene. The breakpoint also separates *OVOL2* from several important downstream CREs predicted to interact with the promoter of the *OVOL2* gene, which could also induce aberrant gene expression.

To exclude the influence of other genes, we analyzed all genes known to be associated with congenital corneal edema. All variants identified were classified as likely benign given the results of their *in silico* analysis and/or, in the case of diseases with autosomal-dominant inheritance, the fact that they were inherited from one of the parents who did not have any signs of corneal endothelial dystrophy.

Currently, regulatory elements that control gene expression are largely unexplored in clinical diagnostics due to interpretive challenges. A future functional investigation to assess the impact of the reciprocal translocation in a spatiotemporal context is required to further validate the hypothesis that ectopic expression of *OVOL2* due to the disruption of *OVOL2* CREs and/or TAD architecture may explain the disease in the proband. As the patient-derived corneal endothelium is inaccessible unless transplantation is required, one way to validate this hypothesis could be the generation of corneal endothelial-like cells derived from induced pluripotent stem cells [11]. However, refinement of such protocols is still required to ensure that a homogenous cell population displaying a lack of *OVOL2* expression can be achieved in the wild-type state.

In summary, this study highlights the need to explore the role of CREs not only in corneal endothelial dystrophies but also in other genetically unsolved human disorders.

## Data Availability

The datasets generated and/or analyzed during the current study are available from the corresponding authors on reasonable request.

## Supplementary Materials

Supplementary Materials Supplementary Table 1: coordinates of the breakpoints in genome builds hg19 and hg38. Supplementary Figure 1: specular microscopy imaging in the proband's left eye. Posterior corneal surface appears uneven with cells that do not have clear borders. Supplementary Figure 2: nucleotide sequences of chromosomal breakpoints and junctions on the derivative chromosomes. Sequences in uppercase are contained in the translocation junctions. One-base microhomology at the junctions is highlighted in yellow. Chromosome regions chr3:149,987,157-149,987,475 (319 bp) and chr20:17,997,229-17,997,374 (145 bp; both in grey and lowercase) are not present on any of the derivative chromosomes. The figure uses hg38 coordinates. Supplementary Figure 3: visualization of topologically associated domains (TADs) at chr20 in H9 ESC-derived neuroectodermal cells. (A) Interaction heat map, in which TADs are indicated as triangles using DI and (B) TopDom algorithms. The position of the breakpoint is highlighted with a red bar. (C) Gene annotations (MANE transcripts), *OVOL2* is shown embedded in a red box. Supplementary Figure 4: visualization of topologically associated domains (TADs) at chr3 in neural progenitor cells. (A) Interaction heat maps, in which TADs are indicated as triangles using DI and (B) TopDom algorithms. The position of the breakpoint is highlighted with a red bar. (C) Gene annotations (MANE transcripts). Supplementary Figure 5: visualization of topologically associated domains (TADs) at chr3 in H9 ESC-derived neuroectodermal cells. (A) Interaction heat map, in which TADs are indicated as triangles using DI and (B) TopDom algorithms. The position of the breakpoint is highlighted with a red bar. (C) Gene annotations (MANE transcripts). Supplementary Table 2: rare heterozygous variants identified in the proband in genes associated with early-onset corneal endothelial dystrophies.
