# Supplementary material for: Disruption of OVOL2 Distal Regulatory Elements as a Possible Mechanism Implicated in Corneal Endothelial Dystrophy
Source: Hum Mutat. 2024 Jan 4;2024:4450082. doi: 10.1155/2024/4450082 (PMC11919061; doi:10.1155/2024/4450082)
Supplement: Supplementary Materials — Supplementary Table 1: coordinates of the breakpoints in genome builds hg19 and hg38. Supplementary Figure 1: specular microscopy imaging in the proband's left eye. Posterior corneal surface appears uneven with cells that do not have clear borders. Supplementary Figure 2: nucleotide sequences of chromosomal breakpoints and junctions on the derivative chromosomes. Sequences in uppercase are contained in the translocation junctions. One-base microhomology at the junctions is highlighted in yellow. Chromosome regions chr3:149,987,157-149,987,475 (319 bp) and chr20:17,997,229-17,997,374 (145 bp; both in grey and lowercase) are not present on any of the derivative chromosomes. The figure uses hg38 coordinates. Supplementary Figure 3: visualization of topologically associated domains (TADs) at chr20 in H9 ESC-derived neuroectodermal cells. (A) Interaction heat map, in which TADs are indicated as triangles using DI and (B) TopDom algorithms. The position of the breakpoint is highlighted with a red bar. (C) Gene annotations (MANE transcripts), OVOL2 is shown embedded in a red box. Supplementary Figure 4: visualization of topologically associated domains (TADs) at chr3 in neural progenitor cells. (A) Interaction heat maps, in which TADs are indicated as triangles using DI and (B) TopDom algorithms. The position of the breakpoint is highlighted with a red bar. (C) Gene annotations (MANE transcripts). Supplementary Figure 5: visualization of topologically associated domains (TADs) at chr3 in H9 ESC-derived neuroectodermal cells. (A) Interaction heat map, in which TADs are indicated as triangles using DI and (B) TopDom algorithms. The position of the breakpoint is highlighted with a red bar. (C) Gene annotations (MANE transcripts). Supplementary Table 2: rare heterozygous variants identified in the proband in genes associated with early-onset corneal endothelial dystrophies. [file 4450082.f1.docx]

**Supplementary Table 1:** Coordinates of the breakpoints in genome builds hg19 and hg38.

|  | **hg19** | **hg38** |
| --- | --- | --- |
| **Break at chromosome 3** | chr3:149,704,943 | chr3:149,987,156 |
|  | chr3:149,705,263 | chr3:149,987,476 |
| **Break at chromosome 20** | chr20:17,977,872 | chr20:17,997,228 |
|  | chr20:17,978,018 | chr20:17,997,374 |


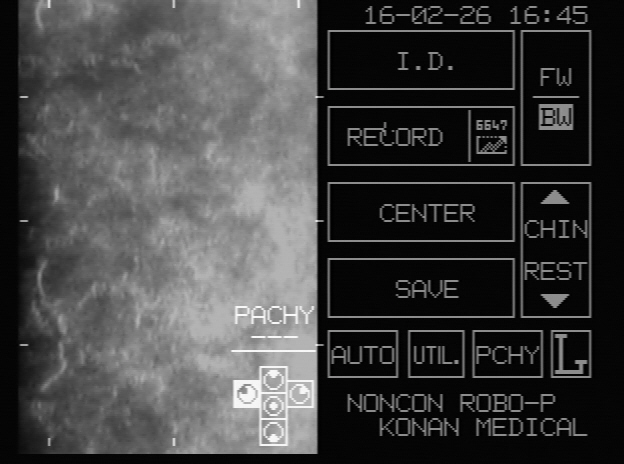


**Supplementary Figure 1:** Specular microscopy imaging in the proband’s left eye. Posterior corneal surface appears uneven with cells that do not have clear borders.

chr3:149987156.

chr3 agcctcctgagtagctgggactacaggtactcaccaccacgcctggctaattttttttttttttgtatatttagtagagacagggtttcaccatgttgccc

der3 junction agcctcctgagtagctgggactacaggtactcaccaccacgcctggctaatagcctctgcctcccaggcacaagtggtcttcccacctcagcctcctgagt

chr20 (reverse) ctccctctgtcccccaggctggagtgaagtggtgtgatcttggctcactgtagcctctgcctcccaggcacaagtggtcttcccacctcagcctcctgagt

.chr20:17997228

chr3:149987476.

chr3 (reverse) gatgatgatgacagctttttattgtctattgtaccagagcctgtggtcctactttgtatattttatatatatattatatagtcctcaaaatagtctttatt

der20 junction gatgatgatgacagctttttattgtctattgtaccagagcctgtggtcctaagtctagactgtcctactgcaccagtctcactcatttctaagctgttgtt

chr20 taaagaacagcaagctaaggtcaactgtgattagaagcattcatttaggcaagtctagactgtcctactgcaccagtctcactcatttctaagctgttgtt

.chr20:17997374

**Supplementary Figure 2**: Nucleotide sequences of chromosomal breakpoints and junctions on the derivative chromosomes. Sequences in uppercase are contained in the translocation junctions. One-base microhomology at the junctions is highlighted in yellow. Chromosome regions chr3:149,987,157-149,987,475 (319 bp) and chr20:17,997,229-17,997,374 (145 bp; both in grey and lowercase) are not present on any of the derivative chromosomes. The figure uses hg38 coordinates.


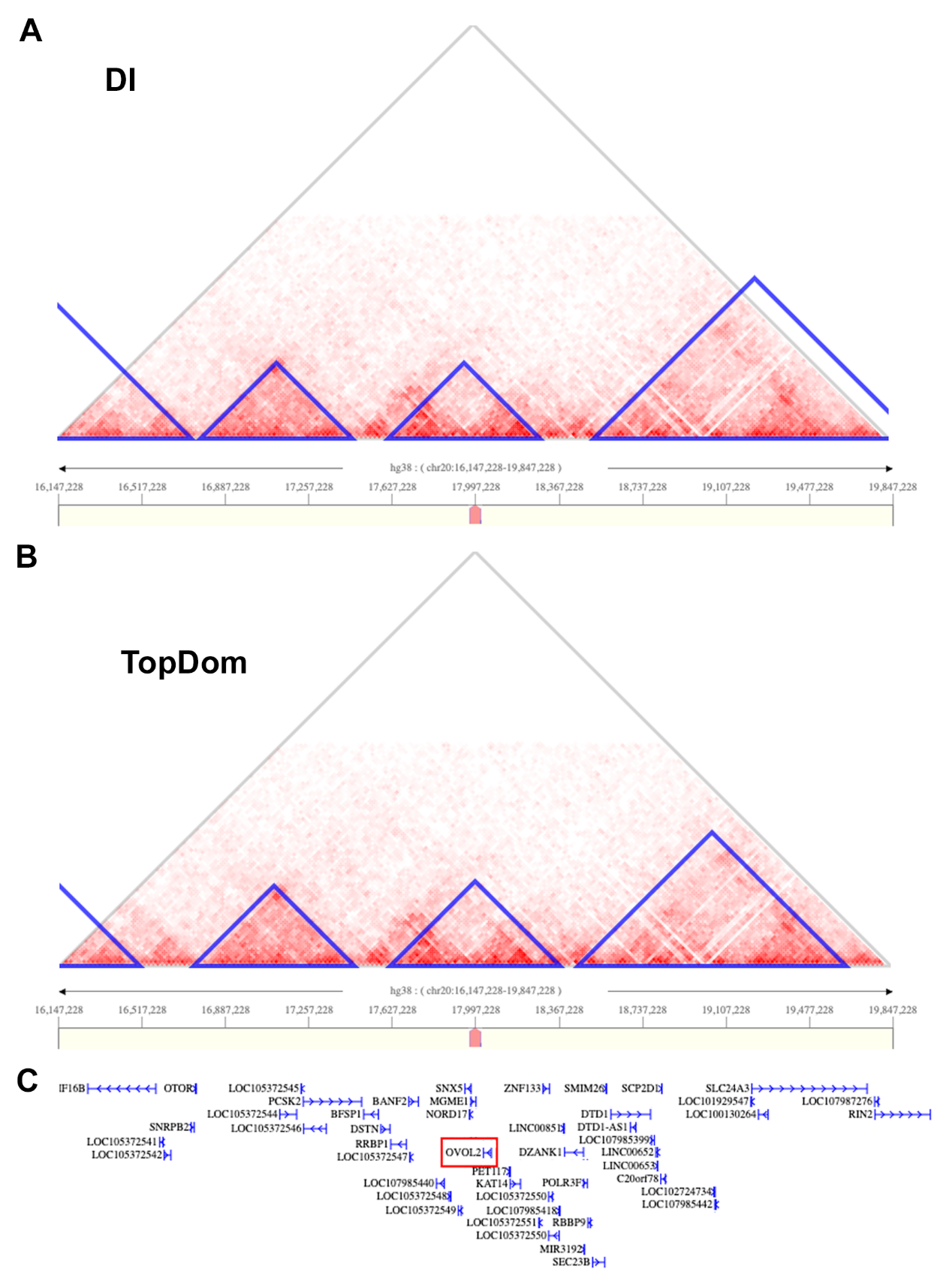


**Supplementary Figure 3:** Visualization of topologically associated domains (TADs) at chr20 in H9 ESC-derived neuroectodermal cells. (A) Interaction heat map, in which TADs are indicated as triangles using DI and (B) TopDom algorithms. The position of the breakpoint is highlighted with a red bar. (C) Gene annotations (MANE transcripts), *OVOL2* is shown embedded in a red box.


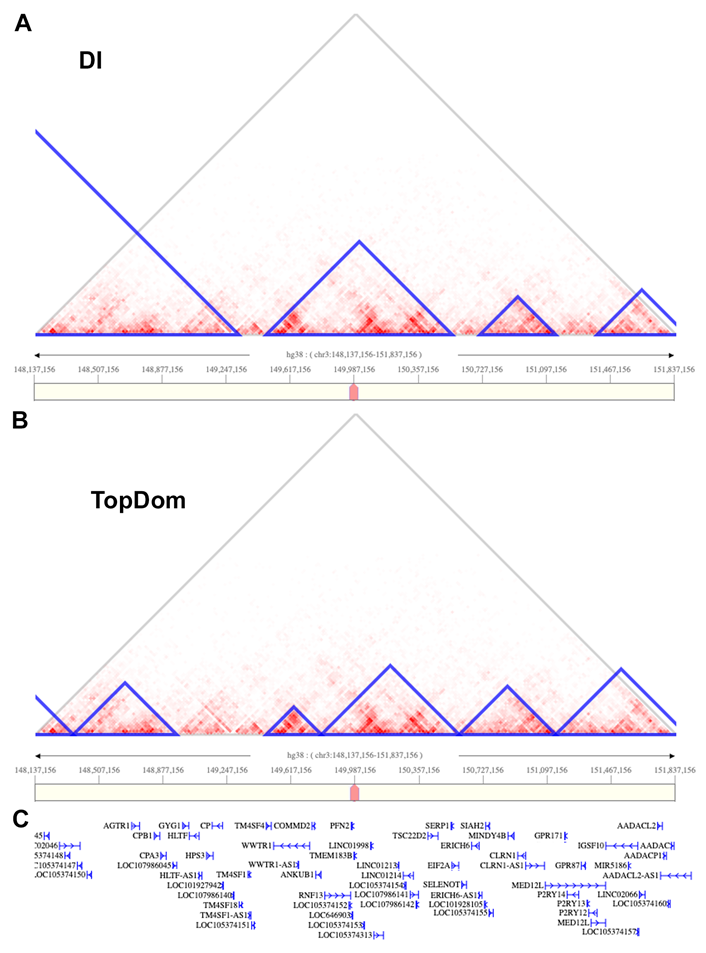


**Supplementary Figure 4:** Visualization of topologically associated domains (TADs) at chr3 in neural progenitor cells. (A) Interaction heat maps, in which TADs are indicated as triangles using DI and (B) TopDom algorithms. The position of the breakpoint is highlighted with a red bar. (C) Gene annotations (MANE transcripts).


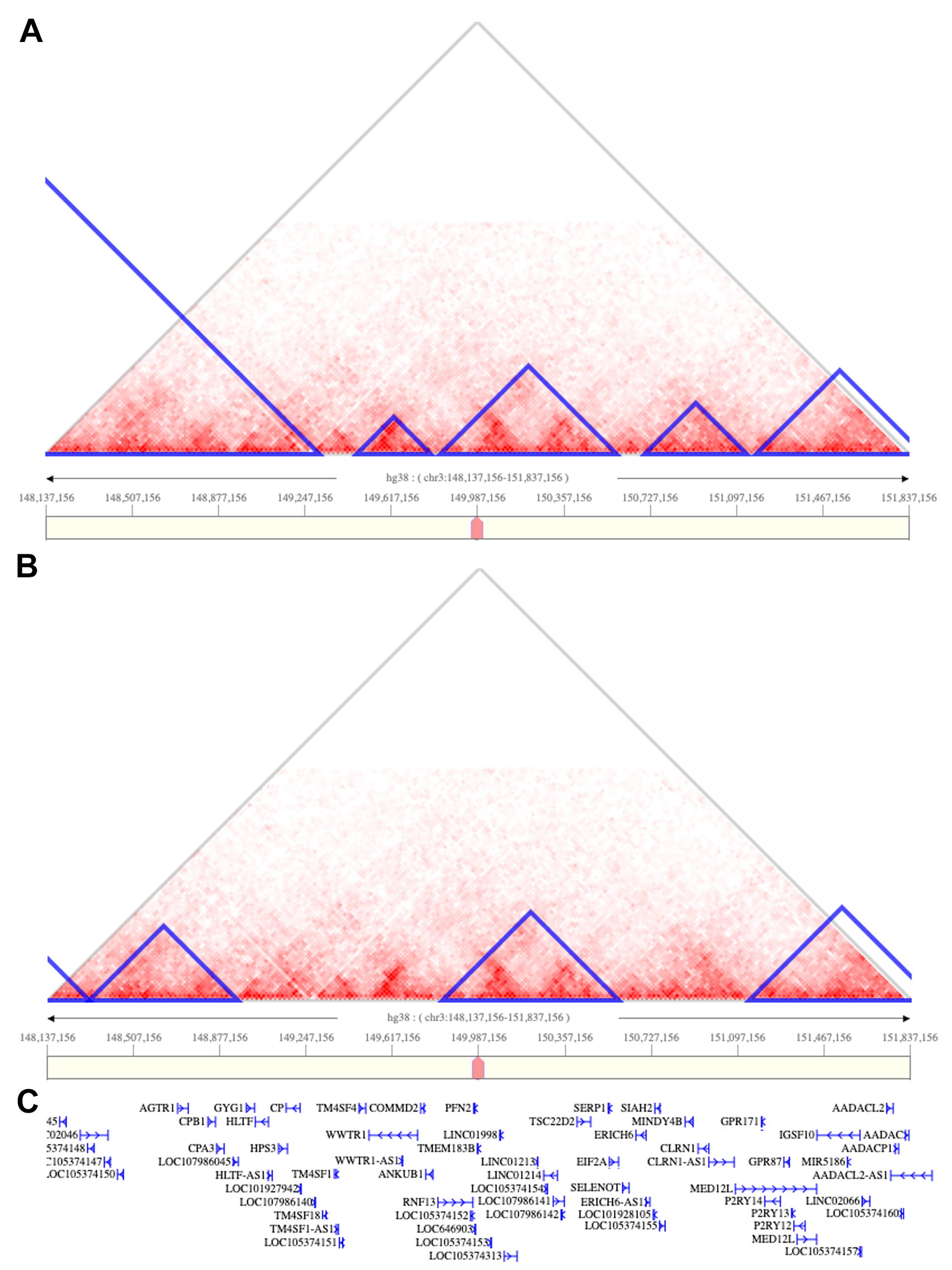


**Supplementary Figure 5:** Visualization of topologically associated domains (TADs) at chr3 in H9 ESC-derived neuroectodermal cells. (A) Interaction heat map, in which TADs are indicated as triangles using DI and (B) TopDom algorithms. The position of the breakpoint is highlighted with a red bar. (C) Gene annotations (MANE transcripts).

**Supplementary Table 2:** Rare heterozygous variants identified in the proband in genes associated with early-onset corneal endothelial dystrophies.

| **hg38** | **hg19** | **Gene name/**  **location** | **Change in the reference sequence** | **Gene and transcript consequences** | **rsID** | **MAF gnomAD**  **v3.1.1** | **Inherited from** |
| --- | --- | --- | --- | --- | --- | --- | --- |
| chr8:101,672,123:T>G | chr8:102,684,351:T>G | *GRHL2*  downstream | c.*5420T>G | None | rs1000368556 | 0.0001188  (18/151,568 alleles) | Mother |
| chr10:31,430,555:A>G | chr10:31,719,484:A>G | *ZEB1*  intronic | c.59-30482A>G | None | none | 0 | Mother |
| chr10:31,423,753:C>A | chr10:31,712,682:C>A | *ZEB1*  intronic | c.59-37284C>A | None | [rs530195180](http://www.ncbi.nlm.nih.gov/projects/SNP/snp_ref.cgi?rs=rs530195180) | 0.0001275  (34/151,984 alleles) | Mother |
| chr10:31,481,989G>T | chr10:31,770,918:G>T | *ZEB1*  intronic | c.260-13790G>T | None | rs1027758472 | 0.000006587  (1/151,818 alleles) | Mother |
| chr20:18,056,439A>G | chr20:18,037,083:A>G | *OVOL2*  intronic | c.321+218T>C | TF binding site | rs1488131108 | 0.000006581  (1/151,952 alleles) | Father |
| chr20:3,236,413T>C | chr20:3,217,059:T>C | *SLC4A11*  intronic | c.88+1131A>G | None | rs973286329 | 0.00001972  (3/152,134 alleles) | *De novo* |

Only variants ≤ 1 Mb from the transcription starts and ends with minor allele frequency ≤ 0.0001 are shown. Reference sequences used: NM_024915.4 (*GRHL2*), NM_030751.6 (*ZEB1*), NM_021220.4 (*OVOL2*), NM_001174089.2 (*SLC4A11*). MAF, minor allele frequency; NA, not applicable; TF, transcription factor. Gene and transcript consequences were derived from Ensembl (<https://www.ensembl.org/Homo_sapiens>).
